# Committed changes in tropical tree cover under the projected 21^st^ century climate change

**DOI:** 10.1038/srep01951

**Published:** 2013-06-06

**Authors:** Zhenzhong Zeng, Shilong Piao, Anping Chen, Xin Lin, Huijuan Nan, Junsheng Li, Philippe Ciais

**Affiliations:** 1 College of Urban and Environmental Sciences, Peking University, Beijing 100871, China; 2 Institute of Tibetan Plateau Research, Chinese Academy of Sciences, Beijing 100085, China; 3 Department of Ecology and Evolutionary Biology, Princeton University, Princeton, N J 08544, USA; 4 State Key Laboratory of Environmental Criteria and Risk Assessment, Chinese Research Academy of Environmental Sciences, Beijing 100012, China; 5 College of Water Sciences, Beijing Normal University, Beijing 100875, China; 6 Laboratoire des Sciences du Climat et de l'Environnement, CEA CNRS UVSQ, 91191 Gif-sur-Yvette, France

## Abstract

Warming and drought pose a serious threat to tropical forest. Yet the extent of this threat is uncertain, given the lack of methods to evaluate the forest tree cover changes under future climate predicted by complex dynamic vegetation models. Here we develop an empirical approach based on the observed climate space of tropical trees to estimate the maximum potential tropical tree cover (MPTC) in equilibrium with a given climate. We show that compared to present-day (2000–2009) conditions, MPTC will be reduced by 1 to 15% in the tropical band under equilibrium future (2090–2099) climate conditions predicted by 19 IPCC climate models. Tropical forests are found to regress or disappear mainly in the current transition zones between forest and savanna ecosystems. This climate pressure on tropical forests, added to human-caused land use pressure, poses a grand challenge to the sustainability of the world's largest biomass carbon pool.

Tropical forest is threatened by global climate changes^1^,^2^ (but see ref. 3) as well as by land use changes induced by increasing food, energy, and development demand^2^,^4^,^5^. Simulations from Dynamic Global Vegetation Models (DGVMs) run with prescribed climate fields, or coupled with General Climate Models (GCMs) consistently indicate that tropical forest, especially the Amazon forest, is likely to be replaced by savanna or C4 grasses in response to projected climate changes^6^,^7^. However, the strength of the climate induced ‘tropical forest dieback' greatly differs among different model simulations^7^,^8^,^9^. This spread of the model results reflects different vegetation – climate relationships emerging from the complex equations of DGVM models. Improving the prediction of future climate-induced loss of tropical forest requires a more quantitative understanding of interactions between vegetation and climate^6^.

In this paper, we quantify the climate envelope of tropical forest by relating tree cover fraction with the observed evapotranspiration (ET). Evapotranspiration through tree crown is one major component of the tropical water balance^10^. In a given climate envelop, by assuming the rate of evapotranspiration through unit area of treeless ground as constant *b*, and that through unit area of tree crown as constant (*a* + *b*), we are able to relate ET and satellite derived tree coverage (TC) with a linear function: *ET* = *a* × *TC* + *b* (1), and to estimate the parameters (*a* + *b*) and *b* which determine the ET demand for a unit tree crown. Note that here our climate envelops are constrained by annual mean air temperature (T) and annual precipitation (P). Radiation (R), which is one of the important factors affecting ET, is not directly included in the climate envelop (see Discussion). Secondly, on decadal scales when runoff and other water storage terms and loss terms can be neglected, in the maximum scenario all water acquired from precipitation (P) can be used for potential tree growth. The climate *maximum potential tree coverage* (MPTC) it can support is thus determined by P and parameters (*a* + *b*) and *b* estimated from Equation (1) (see Methods). This is called potential fraction, because other non-climate factors or indirect climate factors, such as terrain slope, soil fertility, herbivores, disturbance, may further reduce or enhance tree cover^11^, and because human-caused deforestation and degradation will also yield to future forest loss beyond climate effects.

The same parameters of (*a* + *b)* and *b* are also applied to predict future potential MPTC in equilibrium with IPCC climate modeled by the end of the 21^st^ Century (2090–2099)^12^. In addition, atmospheric carbon dioxide (CO_2_) concentration is also projected to rise by the end of this century, which has a profound implication for plant transpiration through decreasing stomatal conductance and increasing water use efficiency. Hence, to estimate the future potential MPTC under rising CO_2_, we introduced to Equation (1) a term of change in stomatal conductance by CO_2_ changes, *δ* (see Methods). The results are also compared with the tree cover fraction simulated by four DGVM ecosystem models (i.e., HYL, LPJ, ORC and TRI)^8^. By using MPTC instead of satellite observed actual tree cover (TC), our aim is to estimate potential MPTC changes that would solely incur from climate limitations, not to project future tree cover, the latter being controlled by natural and anthropogenic factors. We consider instant equilibrium of vegetation response to climate conditions, independent of the pathway and time required for vegetation to reach equilibrium under altered climates^13^.

## Results

**Figure 1:**
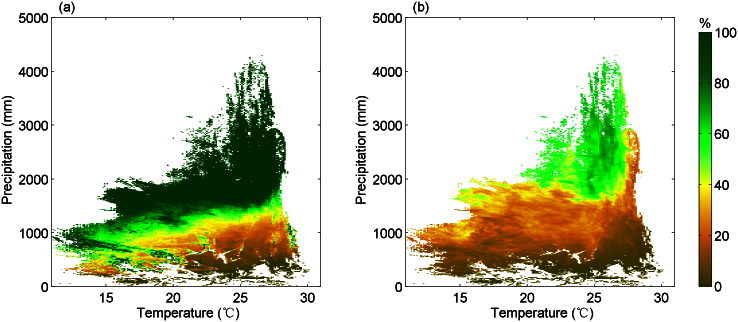
Tropical tree cover fraction in the climate space. (a), The maximum potential tree cover fraction (MPTC). In each climate bin with 0.1°C interval of mean annual temperature and 10 mm interval of annual precipitation, MPTC is estimated by fitting Eq. (1) and Eq. (2) and only shown when the fitting is significant (*p* < 0.05). (b), The MODIS-derived actual tree cover fraction averaged over 2000–2010. Note that the maximum of the MODIS tree cover fraction across the whole tropics is 87% only. The focal area is the tropical vegetation belt between 35°S and 15°N.

Under the condition that trees do not exist where annual rainfall is inferior to evapotranspiration, we estimated the equilibrium MPTC in a (T, P) space discretized in 0.1°C temperature and 10 mm precipitation bins, using gridded fields of T, P and evapotranspiration from satellite observations (see Methods). In 92% of the (T, P) couples, the potential equilibrium MPTC (Fig. 1a) is found to be larger than the actual tree cover fraction observed from space (MODIS tree cover data product; Fig. 1b). This is because factors other than the local water balance reduce the actual tree cover to lower than the potential value^14^,^15^. Oppositely, in a few of the (T, P) climate couples, the actual tree cover exceeds MPTC, which can be caused by, for instance, excessive water from aquifers or from runoff.

**Figure 2:**
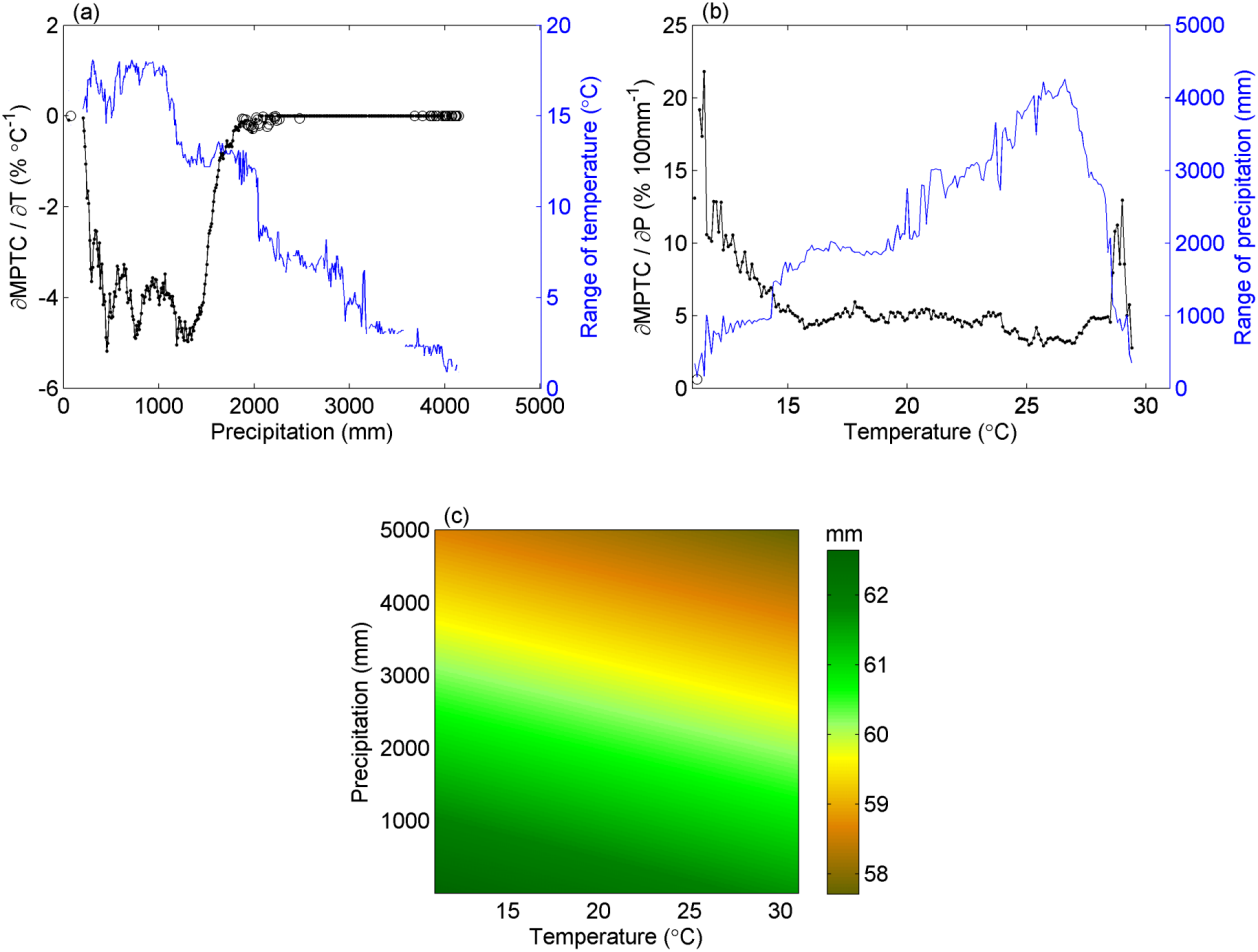
Response of tropical maximum potential tree cover fractions (MPTC) to climate variables. (a), sensitivity of MPTC to mean annual temperature (black) and range of mean annual temperature (blue) along the precipitation gradient. (b), sensitivity of MPTC to annual precipitation (black) and range of annual precipitation (blue) along the temperature gradient. (c), amount of extra precipitation needed to maintain the same MPTC under 1°C warming in the climate space. Pixels are grouped into climate bins with 0.1°C interval of mean annual temperature and 10 mm interval of annual precipitation. Solid dots represent significant (*p* < 0.05) sensitivities while hollow cycles are insignificant ones. The range and the significance together ensure if the calculated sensitivity is meaningful.

Linear regression analyses suggest a significant dependence of both MPTC and TC on P and T (*R^2^* = 0.75, and *R^2^* = 0.72, respectively, Supplementary Table S1). Before it reaches 100% in wet forest areas, MPTC decreases with increasing temperature, and with decreasing precipitation. The sensitivity of MPTC to temperature or precipitation spatial gradients also depends on the other climate variable (Fig. 2). When annual precipitation is below about 1500 mm, the negative sensitivity of MPTC to rising temperature increases with precipitation. On the other hand, the temperature sensitivity of MPTC quickly goes down to zero in regions where precipitation lies in the range 1500–2000 mm yr^−1^, and stays at zero where precipitation reaches above 2000 mm yr^−1^. This is because MPTC saturates to 100% when precipitation is abundant (Figs. 1 and 2). Similarly, the sensitivity of MPTC to spatial precipitation gradients decreases with rising temperature in regions where T > 15°C (Fig. 2b). Fig. 2c shows that the amount of precipitation needed to maintain the same MPTC across a 1°C temperature spatial gradient is roughly of ~60 mm and decreases slightly at higher temperature or precipitation.

**Figure 3:**
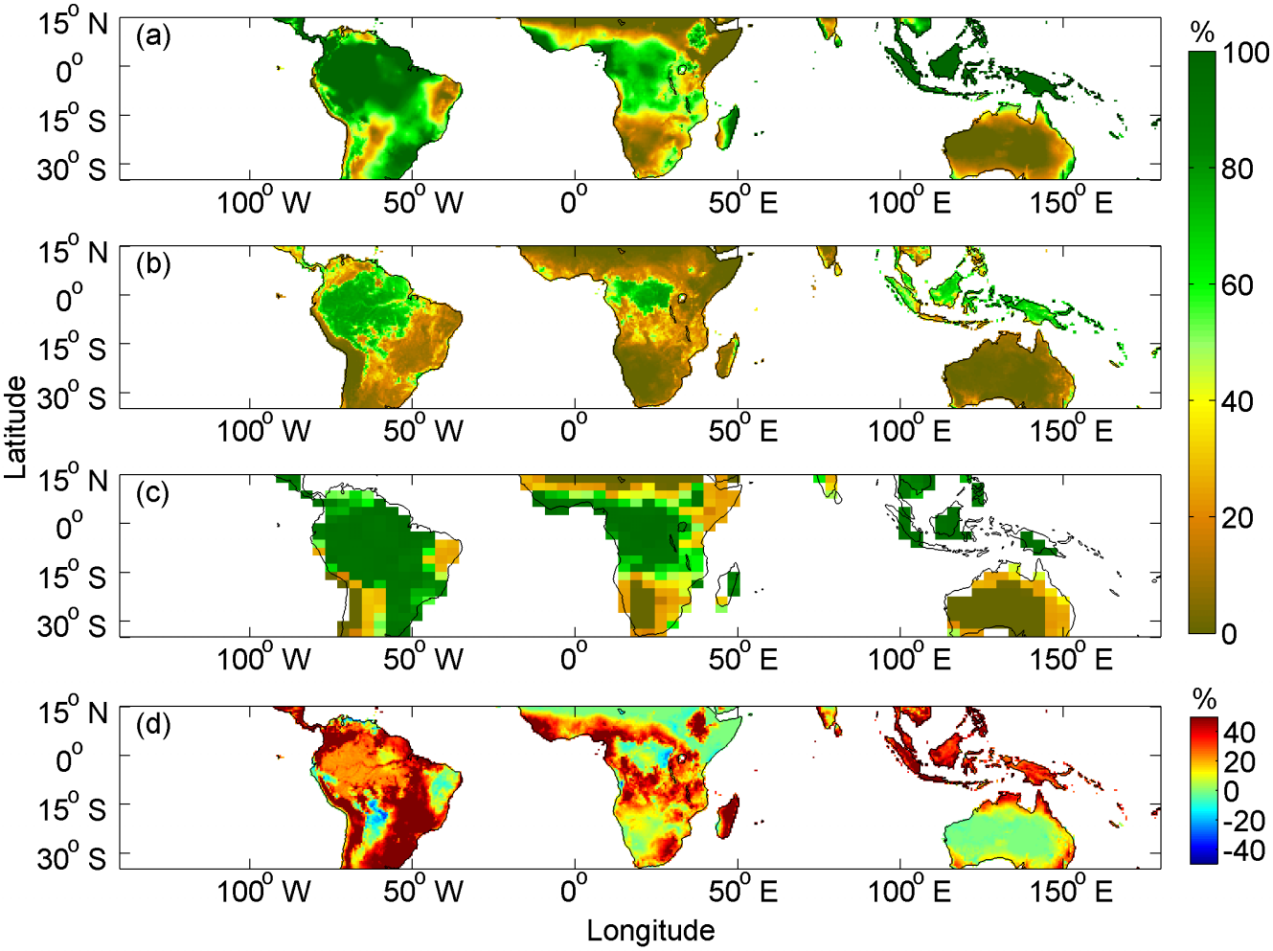
Spatial distribution of multi-year average tree cover fraction during the early 21^st^ century (2000–2009) across the tropics (35°S–15°N). (a), The maximum potential tree cover fraction (MPTC) estimated using present-day climate conditions from CRU datasets. (b), The tree cover fraction derived from MODIS satellite measurements. (c), The multi-model mean tree cover fraction averaged over four DGVMs (i.e., HYL, LPJ, ORC and TRI) under SRES A2. (d), The difference between MPTC and MODIS-derived tree cover fraction across the tropics. Maps were generated using Matlab (http://www.mathworks.co.uk/products/matlab/).

The spatial distribution of MPTC based on empirical regression with T, P predictors, under present-day (2000–2009) climate conditions (see Methods; Fig. 3a) is similar to the tree cover fraction (Fig. 3c) simulated by four DGVM ecosystem models^8^. The DGVM model results also consistently show higher tree cover than the MODIS satellite observed actual tree cover (Fig. 3b), especially in regions currently dominated by C4 grassland and savanna, like in the southeast of South America, around the Congo basin rainforest and Madagascar. In those regions, the overestimation of MPTC can be related to the effects of tree-grass competition, nutrients limitations, fire disturbance that suppresses trees, herbivories, and human caused deforestation^11^,^15^. In the rainforest regions, the discrepancy between potential MPTC and satellite observed actual tree cover fraction is smaller (on average 22% in the rainforest area vs. 34% in the savanna area) (Fig. 3d). Overall, the MODIS observed tree cover is on average 51% only of the potential MPTC (*R* = 0.78, *p* < 0.001).

**Figure 4:**
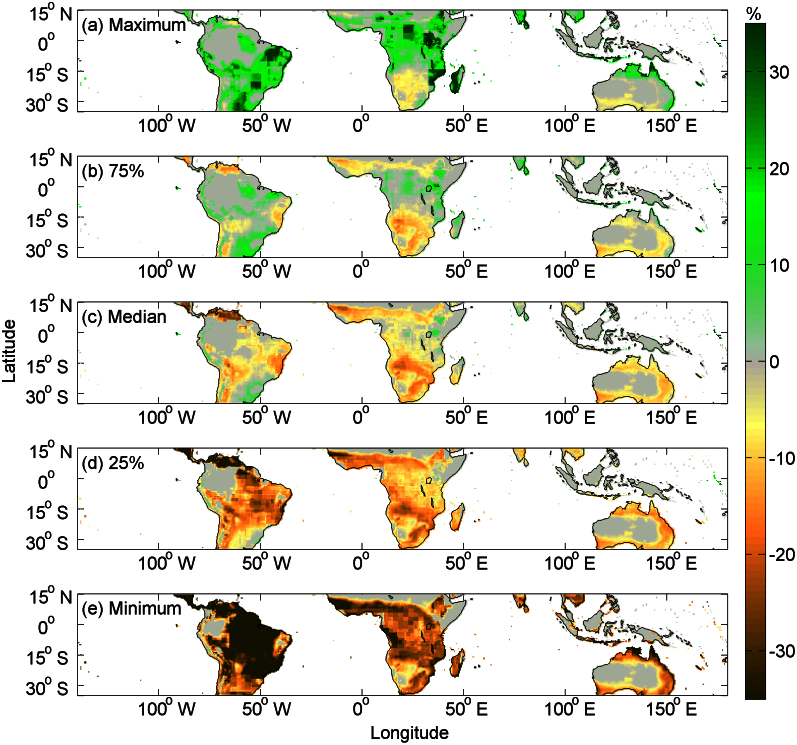
The projected changes in maximum potential tree cover fraction (MPTC) across the tropics over the 21^st^ century under SRES A2. Across the 19 GCMs used in this estimation, projected changes in MPTC between the end of 21^st^ century (2090–2099) and present (2000–2009) are shown for different scenarios in possibility quantiles, including (a), 100% (maximum); (b), 75%; (c), 50% (median); (d), 25%, and (e), 0% (minimum). Maps were generated using Matlab (http://www.mathworks.co.uk/products/matlab/).

For MPTC under the future (2090–2100) climate and CO_2_ scenarios, we used the output of 19 GCMs from the IPCC 4^th^ Assessment Report under the SRES A2 radiative forcing scenario (2090–2100)^16^. The MPTC distribution was found likely to be reduced in most tropical areas under the modeled equilibrium climate conditions of the end of 21^st^ century (2090–2099) (Fig. 4). Figure 4 shows the projected changes in MPTC, relative to present-day values for different scenarios of climate change in possibility quantiles, including 100% (maximum scenario), 75%, 50% (median scenario), 25%, and 0% (minimum scenario). In South America and Africa, the projected future distribution of MPTC varies between different GCM models (Supplementary Fig. S4 and Fig. 4). In Southeast Asia and Australia, MPTC diagnosed from different GCM models exhibits a small spread. Little change of future MPTC is found in Southeast Asia and in Australia. However, there are large uncertainties for the MPTC predictions in South America and Africa, especially in Amazon and central Africa (Fig. 4). In the maximum scenario, MPTC from the ensemble of 19 GCMs indicates that the tropical rainforest in South America and Africa will remain unchanged or even expand (Fig. 4a); while in the minimum scenario, MPTC in the eastern of Amazonia rainforest and Congo rainforest will shrink dramatically (Fig. 4e). Considering all models as indepentent and equally probable, the fraction of climate models that indicate a certain MPTC result can be regarded as a crude metric of the probability for this result^12^. Using this metrics, we infer a high probability (75%, Supplementary Table S2) that the area of the Congo rainforest will be reduced by at least 0.7%, and a medium probability (50%) that the eastern of Amazonia rainforest (extend from 60°W to 48°W) may shrink by at least 5.2% in the end of the 21^st^ century, given climate change. The predicted rainforest dieback in eastern Amazonia is in consistence with the result from Malhi *et al.*^12^ using an empirical preciptation-based boundary reconstruction method, which evaluted the rainfall regime of tropical forest with the 19 GCMs and observed rainfall regime.

The potential tree cover fractions for the four DGVMs under SRES A2 climate vary between models and differ from the empirical MPTC diagnostic using the same GCM of HadCM3 (Supplementary Table S3 and Fig. S5)^8^. With HYL, LPJ and ORC models, the future tree cover in most area of South America and Africa is projected to expand compared to the empirical diagnostic. In TRI, the distribution of future tree cover is similar to that of MPTC under HadCM3 GCM (Supplementary Fig. S5), in which the Congo rainforest will remain mostly unchange, but the Amazonia rainforest will shrink and even disappear, especially in the central Amazon. This overestimated decrease in tree cover from our study compared to DGVMs may be due to the fact that here we only consider the effect of climate alone on vegetation – climate equilibrium; while DGVMs are dynamic models which are not necessary in equilibrium. It has been suggested that the Amazon forest die-back can continue for decades after climate stabilization^13^.

## Discussion

The results of this study suggest that both mean annual precipitation and average surface air temperature are important determinants of tropical tree cover distribution. Recent work focused on precipitation as determinants of tropical vegetation distribution^11^,^12^,^14^,^17^ but ignored temperature because of its homogeneity across the tropics. However, since the temperature in the tropics is also projected to increase steadily and could move away from optimum for tree growth during this century^1^,^3^, its role as a determinant of tropical vegetation distribution cannot be ignored in evaluating future vegetation shift induced by climate changes, which is evidenced by our sensitivity analysis of potential tree cover to climate factors. Temperature regulates tropical tree cover mainly through its control on plant transpiration. It is found that along the temperature gradient, the plant transpiration parameter (*a* + *b*) varies remarkably, while the evaporation parameter *b* remains roughly constant (Supplementary Fig. S8).

Despite of the high probability of decreasing tree cover fraction across most area of the tropics including the eastern of Amazonia and Congo rainforest under equilibrium future climate conditions, our empirical MPTC results may still underestimate the extent of tropical forest dieback in response to climate change, especially in the forest – savannah transition areas. In our static empirical model, the equilibrium response of tree cover to temperature and precipitation is linear. However, it may not be the case over the forest – savannah transition areas where tree – grass competition, fire and herbivory disturbances could bring rapid and nonlinear vegetation shift from forested land to savannah in response to increasing temperature or decreasing precipitation^18^,^19^. Climate defined MPTC is 1.61 times of that of satellite observed tree cover fraction in those transition areas where tree cover is about 0.50 ~0.60; while it is only 1.29 times of observed tree cover fraction in forested lands. Higher level of land conversion in the forest – savannah transition areas with more human dwelling than in forested lands may also contribute to its higher reduction in tree cover from the climate maximum values.

Our findings highlight the important role of temperature, precipitation, as well as atmospheric CO_2_ in determining tropical tree coverage. Yet our results should be viewed as the outcome of a particular set of assumptions, rather than an assertion on the future change in tropical tree cover. Because our empirical approach only considers the effects of precipitation, temperature and atmospheric CO_2_ concentration, the results are subjected to a certain degree of uncertainty. For example, tropical vegetation distribution is also significantly associated with the temporal (seasonal, interannual) distribution of rainfall^17^. Disturbance regimes such as fire, grazing and human intervention also play important roles on the potential tree cover^11^,^18^. Moreover, it has been well documented that net radiation affects both transpiration rate and evaporation rate^20^,^21^. Yet because of the lack of high spatial resolution dataset of radiation, particularly the unknown change in future radiation, we could not include net radiation in quantifying the future changes in MPTC. By defining the ET-TC relationship only in a (T, P) space, we have assumed that the future changes in temperature and radiation can be synchronized. Yet the future warming may not be accompanied with increased radiation. Thus, it is likely that the warming induced future ET demand for a unit area of tree crown may be overestimated, which consequently results in the underestimated MPTC. The range of this uncertainty, however, is difficult to assess, since the change in future radiation, especially the short wave radiation, as well as the relative dependency of ET upon radiation after accounting for that upon temperature, is unknown. In addition, in a region where water lost through runoff is sizable, assuming zero runoff overestimates the potential tree cover. In fact, runoff can also be described as a function of TC in a given climate envelope as vegetation system can reduce water loss from runoff^22^,^23^,^24^. However, the lack of high resolution (i.e. 1 km) data of global runoff prevents us from exploring on the relationship between runoff and TC. Further experiments and analyses, in particular those based on high spatial resolution runoff and net radiation datasets, are needed to explore the determinant factors of tree cover and their mechanisms.

## Methods

### Datasets

We focused on the tropical vegetation between 35°S and 15°N, including Africa, Australia, South Asia and South America^18^, which were gridded at the scale of 1 km^2^. The grids were grouped into climatic bins with resolutions of 10 mm of P (ranging from 0 to 5010 mm) and 0.1°C of T (ranging from 11 to 31°C). Data used in this study include satellite observed tree cover fraction (TC), mean annual evapotranspiration (ET), mean annual precipitation (P) and average surface air temperature (T) at the resolution of 1 km^2^. Satellite observed tree cover fraction was computed from 0.25 km resolution MOD44B Collection 005 production from 2000 to 2010, deriving from Moderate Resolution Imaging Spectroradiometer (MODIS) satellite measurement of canopy reflectance^25^. Multiyear mean annual evapotranspiration from 2000 to 2010 were extracted from MOD16 production (MOD16 ET) at 1 km resolution, which is computed globally every day using MODIS land cover, FPAR/LAI data and global surface meteorology from the Global Modeling and Assimilation Office (GMAO)^21^. Both multiyear mean annual precipitation and average surface air temperature were obtained from WorldClim at 1 km resolution based on meteorological station data from 1950–2000^26^. Observed multiyear mean values of P and T for the early 21^st^ century (2000–2009) were obtained from the Climate Research Unit (CRU) TS3.1 datasets at the resolution of 0.5° × 0.5°^27^.

The late 21^st^ century (2090–2099) climate is the sum of current climate and current climate multiplied by the relative climate changes^12^, which is estimated using all the 19 Global Climate Models (GCM) in the Intergovernmental Panel on Climate Change (IPCC) AR4 under the medium-high range Special Report on Emissions Scenarios (SRES) A2^16^ (https://esg.llnl.gov:8443/index.jsp). The four DGVMs^8^ used in this study are the HyLand model (HYL), the Lund-Potsdam-Jena model (LPJ), ORCHIDEE model (ORC) and TRIFFIED model (TRI). We don't include Sheffied model since vegetation in this model is fixed^8^. All of these models were coupled to a GCM analogue model and a simple ocean carbon cycle model IMOGEN, Integrated Model Of Global Effects of climatic aNomalies, calibrated against the climate change simulated by HadCM3LC under four SRES^8^.

### Analyses

The estimation of MPTC is based on the following assumptions. Firstly, we only consider evapotranspiration conducted through tree crown which is thus proportional to tree cover fraction^28^,^29^, (e.g. Supplementary Fig. S1) 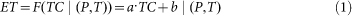
where ET is mean annual evapotranspiration, TC is average tree cover fraction, P is mean annual precipitation and T is average surface air temperature. Note parameter *a* should be positive and regressions with negative *a* are not included in the following analyses (see Supplementary Fig. S2).

We fitted Equation (1) for each climatic bin of specific P and T with the satellite observed TC and climate data when its sampling size is larger than 100. When runoff is neglected, under the maximum potential, evapotranspiration through tree crown would balance the precipitation it receives. Hence, the climate defined maximum potential tree cover fraction (MPTC) is the tree cover fraction that makes ET equal to P, 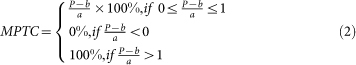
where *a* and *b* are least-squares fitted parameters derived from Equation (1).

Here MPTC are estimated for each climatic bin of specific P and T. It is treeless when MPTC is 0, and fully forested when MPTC is 100%. The states of treeless or fully forested are not sensitive to small changes in climate variables as shown in Fig. 2.

On the other hand, the shifts of vegetation in response to the climate change may be partly mitigated by the rising atmospheric CO_2_ concentration, which is predicted to rise to 730–1020 ppm by 2100 under SRES A2^9^. Under higher CO_2_ pressure, leaf stomata open less to reduce water loss while uptaking the same amount of CO_2_, which results in enhanced water use efficiency^2^,^12^. Thus, without considering possible changes on surface energy balance, the future ET under rising atmospheric CO_2_ can be expressed as a function of changes in stomatal conductance^23^: 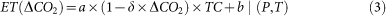
where *ET*(Δ*CO*_2_) is the ET value when atmospheric CO_2_ concentration increased by Δ*CO*_2_, TC is tree cover, *δ* is the relative change in stomatal conductance caused by per ppm increase in CO_2_, *a* and *b* are least-squares fitted parameters derived from Equation (1). Here Δ*CO*_2_ by the end of 21^st^ century and *δ* over the tropics are assumed as 500 ppm^9^ and 0.03% per ppm^12^,^30^,^31^, respectively. Thus the relative changes in stomatal conductance caused by rising CO_2_ (*δ* × Δ*CO*_2_) is 15%.

Below we denote the future MPTC under rising atmospheric CO_2_ as MPTC', which can be calculated as: 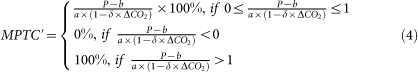
where MPTC' is the maximum potential tropical tree cover under increased atmospheric CO_2_ concentration, *a* and *b* are least-squares fitted parameters derived from Equation (1).

Finally, we fit linear regression models of MPTC and MPTC' as a function of P, T, and their product, with the least-square estimation method, when MPTC or MPTC' falls between 0 and 100% (see Supplementary Fig. S3), 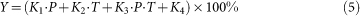
where Y is MPTC or MPTC', P is mean annual precipitation and T is average surface air temperature, *K_1_*, *K_2_*, *K_3_*, *K_4_* are constants.

## Supplementary Material

Supplementary Information Supporting Information
