# Supplementary material for: Committed changes in tropical tree cover under the projected 21st century climate change
Source: Sci Rep. 2013 Jun 6;3:1951. doi: 10.1038/srep01951 (PMC3674425; doi:10.1038/srep01951)

Supplementary Information

Committed changes in tropical tree cover under the projected 21st century climate change

Zhenzhong Zeng, Shilong Piao, Anping Chen, Xin Lin, Huijuan Nan, Junsheng Li, Philippe Ciais

Supplementary Table S1 Regression coefficients in Eq. (5) (see Detailed Methods). The linear models are developed for the maximum potential tree cover (MPTC), MPTC' (MPTC under 500 ppm increase in atmospheric CO_2 concentration), and MODIS-derived average, maximum, minimum, and median tree cover (TC) within climatic bins, respectively.

dependent variables (%)	coefficients				R^2	P
	intercept	P (10^{-4} mm)	T (10^{-2} ℃)	P·T (10^{-6} mm·℃)		
MPTC	0.669	5.67	-3.59	0.459	0.75	<0.001
MPTC'	0.743	6.34	-3.88	-0.426	0.76	<0.001
MODIS average TC	0.0105	3.20	-0.170	-5.72	0.72	<0.001
MODIS maximum TC	0.692	3.23	-1.20	-2.22	0.42	<0.001
MODIS minimum TC	-0.0166	0.510	0.0410	-1.51	0.56	<0.001
MODIS median TC	-0.0173	2.10	-0.0248	-3.02	0.56	<0.001

Supplementary Table S2 Projected changes in maximum potential tree cover (MPTC) over the 21st century in the Amazon Basin, Congo Basin, South Asia (90°E-160°E, 10°S-15°N) and the whole tropics (35°S-15°N) under SRES A2. Across the 19 GCMs used in this estimation, projected changes in MPTC between the end of 21st century (2090-2099) and present (2000-2009) are given for different scenarios in 100% (maximum), 75%, 50% (median), 25%, and 0% (minimum) quantiles, respectively. The unit is %.

	Amazonia	Congo	South Asia	Tropics
Maximum	2.6	7.8	2.0	-0.9
75%	1.0	-0.7	0.2	-2.9
Median	-2.4	-4.7	-0.1	-5.5
25%	-10.6	-7.5	-1.1	-7.9
Minimum	-38.1	-16.3	-4.1	-14.8

Supplementary Table S3 Projected changes in DGVM-simulated tree cover between the early 21st century (2000-2009) and the late 21st century (2090-2099) in the Amazon Basin, Congo Basin, South Asia (90°E-160°E, 10°S-15°N) and the whole tropics (35°S-15°N) under SRES A2. Four DGVMs are used to estimate the projected changes in tree cover, including the HyLand model (HYL), Lund-Potsdam-Jena model (LPJ), ORCHIDEE model (ORC), and TRIFFIED model (TRI). All DGVMs are coupled to a GCM HadCM3. The projected changes in maximum potential tree cover (MPTC) using climate projections of HadCM3 are also given in this table. The unit is %.

	Amazonia	Congo	South Asia	Tropics
HYL	-11.8	0.7	0.1	2.6
LPJ	-3.7	1.7	0.1	5.1
ORC	-5.4	1.3	1.6	4.4
TRI	-48.3	-1.4	0.7	-7.5
This Study	-38.1	-3.8	-1.2	-14.8

Supplementary Figure S1 The relationship between MODIS-derived mean annual evapotranspiration (ET) and tree cover fraction (TC). Each point represents a pixel in a climatic bin with mean annual temperature (T) interval of 25.15–25.25 ℃ and annual precipitation (P) interval of 1000–1010 mm. In this climatic bin, ET is proportional to TC (R^2=0.66, p<0.01).

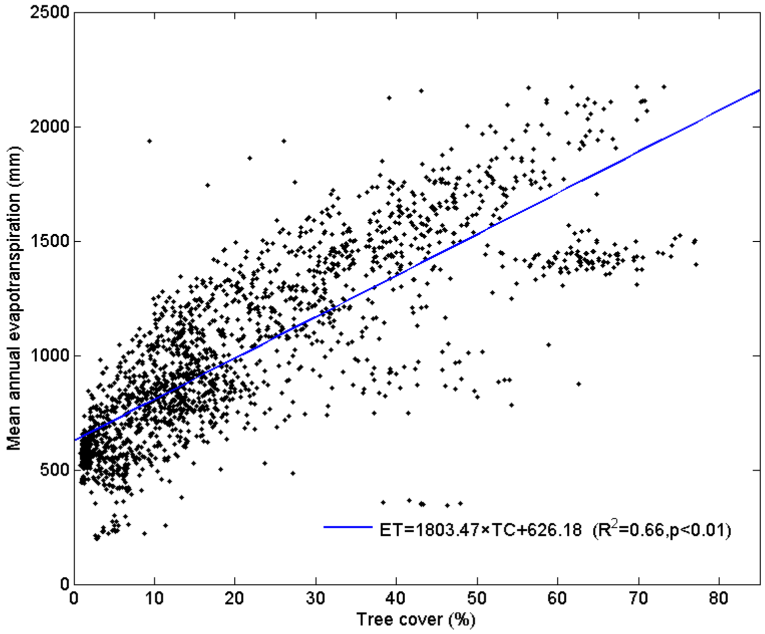

Supplementary Figure S2 The relationship between MODIS-derived mean annual evapotranspiration (ET) and tree cover fraction (TC). Each point represents a pixel in a specific climatic bin with (a) mean annual temperature (T) interval of 25.45–25.55 ℃ and annual precipitation (P) interval of 2820–2830 mm; (b) T interval of 25.55–25.65 ℃ and P interval of 2820–2830 mm. Although with similar climate conditions, the linear regression models between ET and TC for the two climatic bins are different. Note that for **b**, the slope of the linear model is negative, therefore the climatic bin should be excluded in the following analyses.

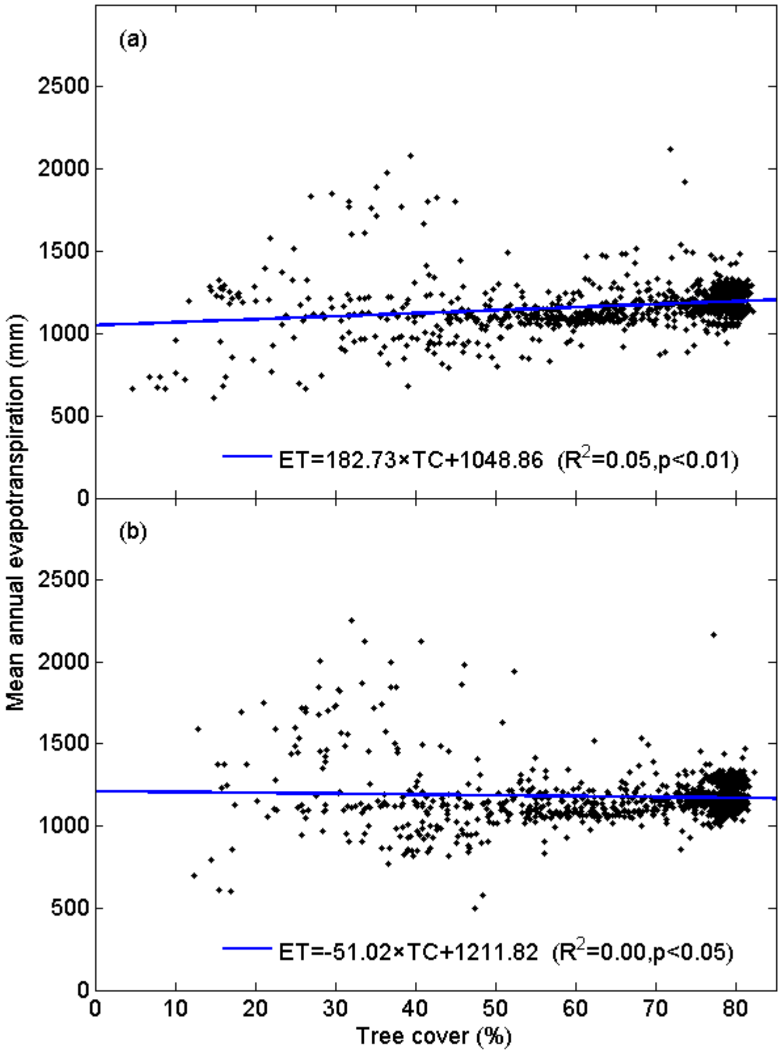

Supplementary Figure S3 The tropical maximum potential tree cover fraction (MPTC) in the climate space. (a), MPTC estimated using Eq. (5). (b)**,** MPTC estimated using Eq. (1) and Eq. (2). For climatic bins without MPTC estimates, MPTC estimated from Eq. (5) using corresponding temperature and precipitation are shown. The climate space for the focal area is defined by climate bins with 0.1 ℃ interval of mean annual temperature (ranging from 11 to 31℃) and 10 mm interval of annual precipitation (ranging from 0 to 5010 mm).

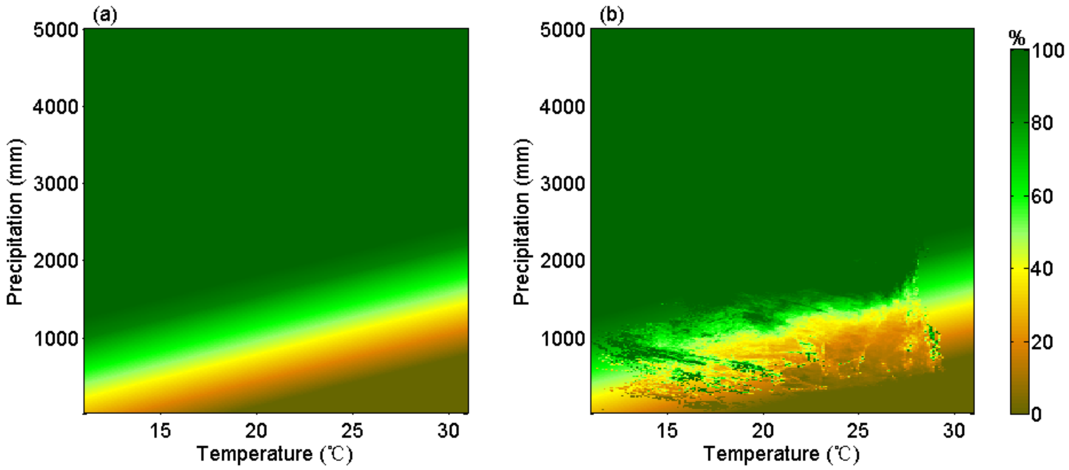

Supplementary Figure S4 Projected maximum potential tree cover fraction (MPTC) and DGVM-simulated tree cover fraction across the tropics by the end of the 21st century under SRES A2. Across the 19 GCMs used, projected MPTC during 2090-2099 is shown for different scenarios in possibility quantities, including (a) 100% (maximum); (c) 75%; (e) 50% (median); (g) 25%; and (i) 0% (minimum). Simulated tree cover fractions during 2090-2099 are derived from four DGVMs coupled to a GCM HadCM3: (b) HYL; (d) LPJ; (f) ORC; (h) TRI. The projected MPTC using climate projections of HadCM3 is also shown in Fig. j.

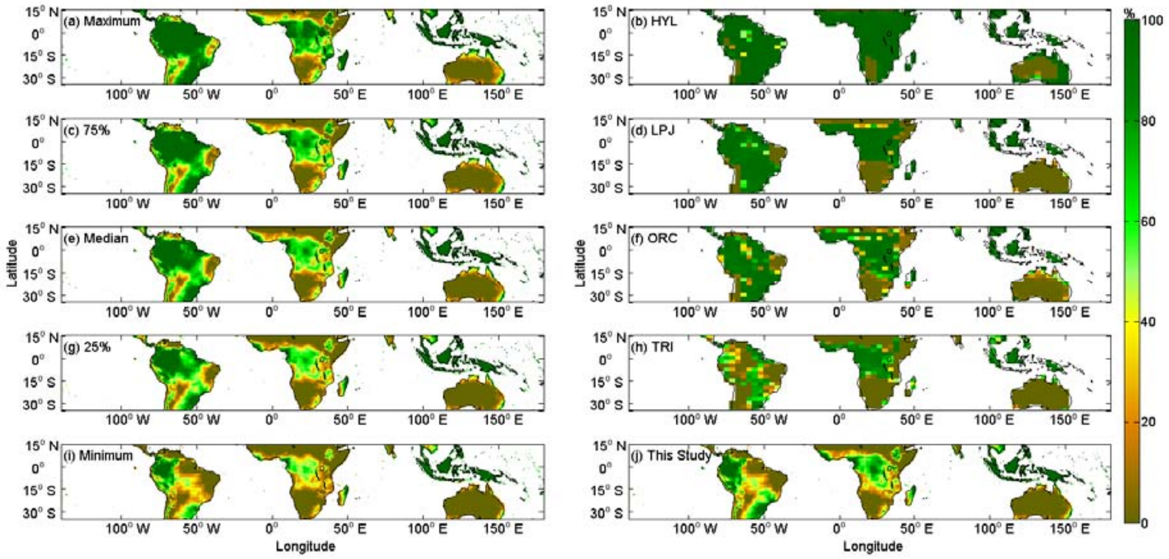

Supplementary Figure S5 The changes in DGVM-simulated tree cover across the tropics over the 21st century under SRES A2. The difference in simulated tree cover fractions between the end of 21st century (2090-2099) and present (2000-2009) are derived from four DGVMs coupled to a GCM HadCM3: (a) HYL; (b) LPJ; (c) ORC; (d) TRI. The projected change in tropical MPTC between the two periods using climate projections of HadCM3 is also shown in Fig. e.

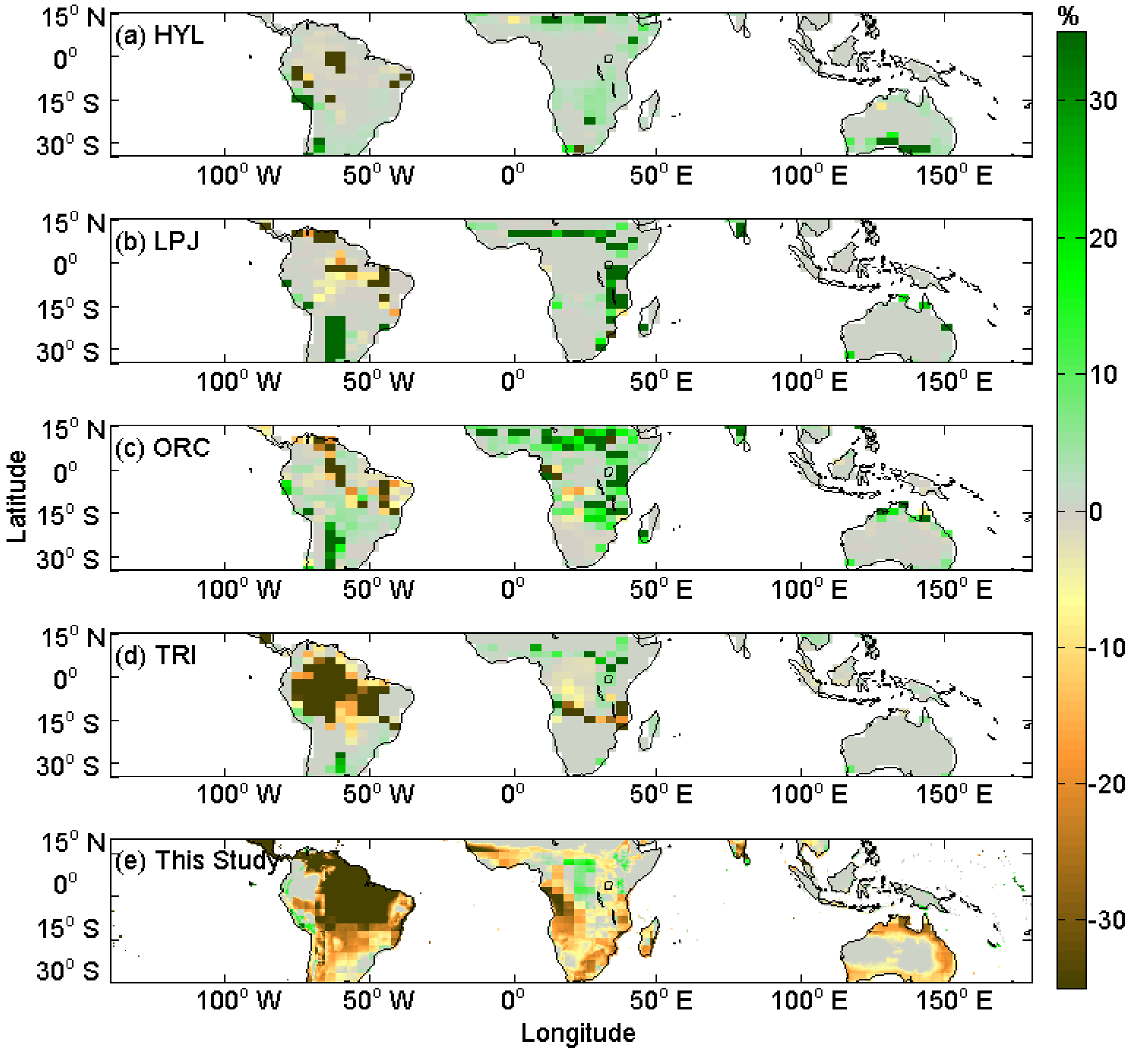

Supplementary Figure S6 The projected maximum potential tree cover fraction (MPTC) and changes in MPTC across the tropics over the 21st century under SRES A1B. Across the 24 GCMs used, projected MPTC by the end of 21st century (2090-2099) (the left panels) and changes in MPTC compared to present-day conditions (2000-2009) (the right panels) are shown for different scenarios in possibility quantities, including a-b, 100% (maximum), c-d, 75%, e-f, 50% (median), g-h, 25%, and i-j, 0% (minimum).

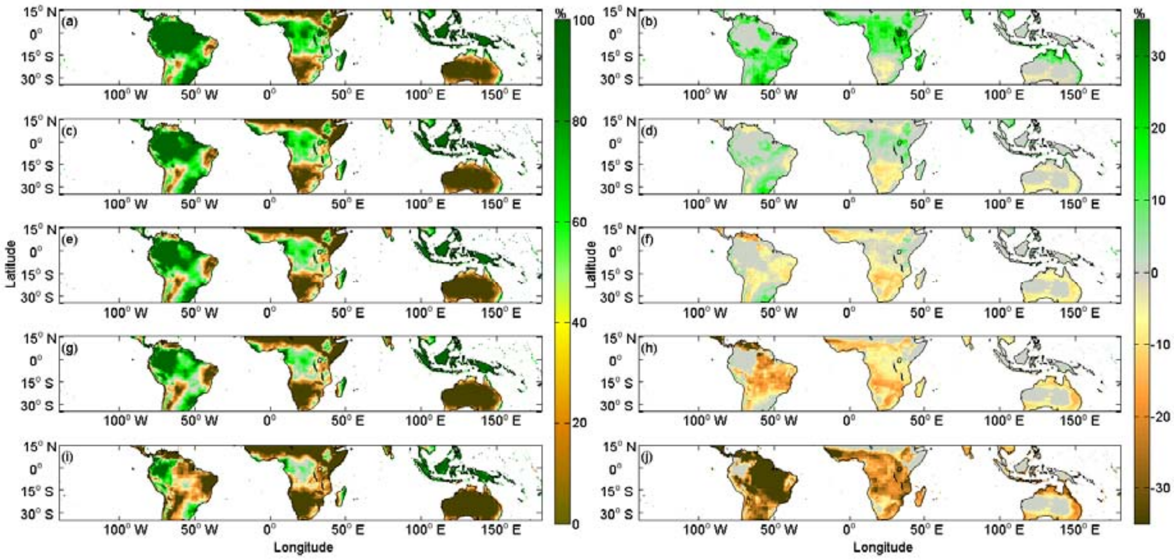

Supplementary Figure S7 The projected maximum potential tree cover fraction (MPTC) and changes in MPTC across the tropics over the 21st century under SRES B1. Across the 21 GCMs used, projected MPTC by the end of 21st century (2090-2099) (the left panels) and changes in MPTC compared to present-day conditions (2000-2009) (the right panels) are shown for different scenarios in possibility quantities, including a-b, 100% (maximum), c-d, 75%, e-f, 50% (median), g-h, 25%, and i-j, 0% (minimum).

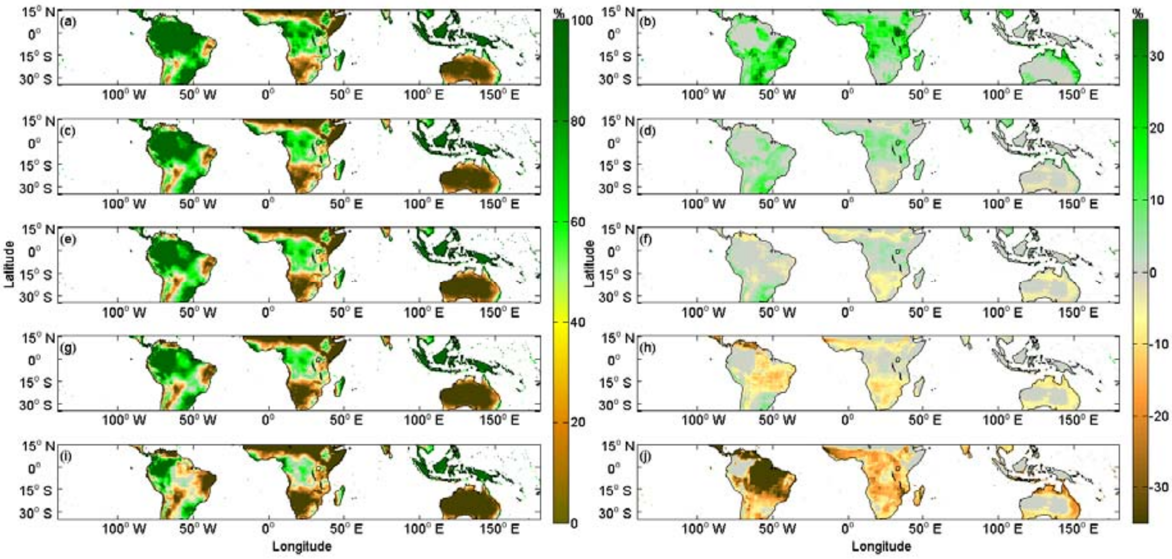

Supplementary Figure S8 Parameters of Equation (1) in climate space. (a), parameter *b*, which is the unit area evaporation rate. (b), parameter (*a*+*b*) which is the unit area plant transpiration rate. The parameters are estimated by fitting Equation (1) for each climate bin with 0.1 ℃ interval of mean annual temperature and 10 mm interval of annual precipitation, and only those of significant ($p<0.05$) fitting are shown.

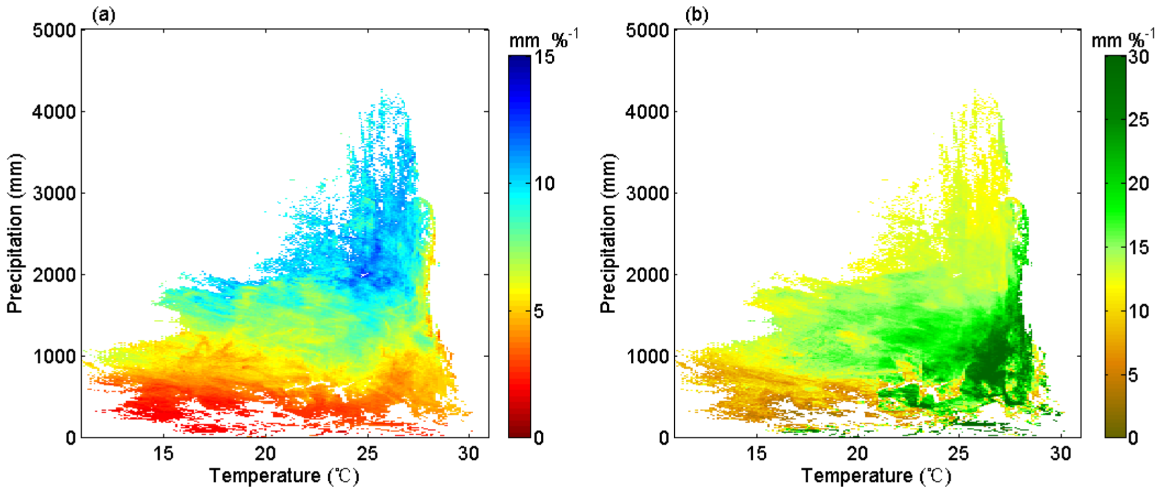

Supplement: Supplementary Information — Supporting Information [file srep01951-s1.pdf]
